# Atmospheric Anæsthesia

**Published:** 1877-05

**Authors:** M. J. Eley


### ARTICLE IX.

### *Atmospheric Anæsthesia.*

If your readers desire the benefits of this unique plan of avoiding pain in minor surgery, let them read, and profit from, the following cases, the last two of many I have met:

1. R. W., male, aged thirty, complaining of felon on finger, which needs lancing. I ordered rapid and deep respiration, and after two minutes plunged the knife to the bone, making a full and free incision. Result, a slight twinge of pain.

2. Benjamin S.; complains of a decayed, aching molar. I directed the respiration, as before, and extracted with some difficulty. The patient, a stout young man, asked, "did you get it," declaring he felt no pain whatever, whereas he complained considerably before, in extracting a smaller tooth.

I have tried many cases thus with success, occasionally failing;. I fully believe in the latter, because I could not obtain the requisite rapidity and deepness in respiration.

I have also tried, with success, the rapid and deep respiration of chloroform, permitting only two or three respirations, and operating immediately, as in the above cases.

—*Med. and Surg. Reporter.* M. J. Eley, M. D.

---

